# T-Type Calcium Channel Inhibitors Induce Apoptosis in Medulloblastoma Cells Associated with Altered Metabolic Activity

**DOI:** 10.1007/s12035-022-02771-0

**Published:** 2022-03-04

**Authors:** Mohammed Sedeeq, Ahmed Maklad, Taush Dutta, Zikai Feng, Richard Wilson, Nuri Gueven, Iman Azimi

**Affiliations:** 1 School of Pharmacy and Pharmacology, College of Health and Medicine, University of Tasmania, Hobart, Tasmania 7001 Australia; 2 Central Science Laboratory, University of Tasmania, Hobart, Tasmania 7001 Australia

**Keywords:** Medulloblastoma, T-type calcium channels, Mibefradil, NNC-55–0396, Metabolic activity, Apoptosis

## Abstract

Medulloblastoma (MB) is the most common malignant paediatric brain tumour. In our previous studies, we developed a novel 3D assay for MB cells that was used to screen a panel of plasma membrane calcium channel modulators for their effect on the 3D growth of D341 MB cells. These studies identified T-type (CaV3) channel inhibitors, mibefradil and NNC-55–0396 (NNC) as selective inhibitors of MB cell growth. Mibefradil was originally approved for the treatment of hypertension and angina pectoris, and recently successfully completed a phase I trial for recurrent high-grade glioma. NNC is an analogue of mibefradil with multiple advantages compared to mibefradil that makes it attractive for potential future clinical trials. T-type channels have a unique low voltage-dependent activation/inactivation, and many studies suggest that they have a direct regulatory role in controlling Ca^2+^ signalling in non-excitable tissues, including cancers. In our previous study, we also identified overexpression of CaV3.2 gene in MB tissues compared to normal brain tissues. In this study, we aimed to characterise the effect of mibefradil and NNC on MB cells and elucidate their mechanism of action. This study demonstrates that the induction of toxicity in MB cells is selective to T-type but not to L-type Ca^2+^ channel inhibitors. Addition of CaV3 inhibitors to vincristine sensitised MB cells to this MB chemotherapeutic agent, suggesting an additive effect. Furthermore, CaV3 inhibitors induced cell death in MB cells via apoptosis. Supported by proteomics data and cellular assays, apoptotic cell death was associated with reduced mitochondrial membrane potential and reduced ATP levels, which suggests that both compounds alter the metabolism of MB cells. This study offers new insights into the action of mibefradil and NNC and will pave the way to test these molecules or their analogues in pre-clinical MB models alone and in combination with vincristine to assess their suitability as a potential MB therapy.

**Supplementary Information:**

The online version contains supplementary material available at 10.1007/s12035-022-02771-0.

## Introduction

Medulloblastoma (MB) is a primary malignant tumour of the cerebellum that is rarely seen in adults and predominantly occurs in children. Indeed, MB is the most common childhood malignant brain tumour, representing 20% of all paediatric brain cancers [1]. Current treatment options include a combined-modality approach of surgical resection, radio- and chemotherapy. Although these approaches improve the survival of MB patients, they are not completely effective, in particular for high-risk MB. In addition, these treatment modalities are associated with adverse events and severe treatment-related morbidities [2]. Hence, new treatment options are urgently needed.

MB is categorised into four molecular subgroups: wingless (WNT), sonic hedgehog (SHH), group 3 and group 4. Groups 3 and 4 tumours are associated with higher levels of metastasis and worse clinical outcomes than the other two groups [1]. Calcium signalling critically affects many cancer hallmarks, including cell proliferation, apoptosis resistance, metastasis and angiogenesis [3]. Altered expression and/or activity of plasma membrane calcium channels have been reported in several types of cancers, and pharmacological modulators of specific Ca^2+^ influx channels have been proposed to represent potential future agents for cancer therapy [4]. T-type channels have a unique low voltage-dependent activation/inactivation which indicate that they may be particularly effective in controlling intracellular Ca^2+^ oscillations [5]. Additionally, many studies suggest that these channels have a direct regulatory role in controlling Ca^2+^ signalling in non-excitable cells, including some cancerous cells [5–7]. In our previous study, we analysed calcium signalling regulating genes in MB patients using publicly available datasets. This study showed that CACNA1H (CaV3.2) gene was significantly upregulated in MB tissues compared to normal brain tissues, and its levels were associated with cancer metastasis and patients survival rates [8]. This study suggested the potential importance of this Ca^2+^ channel in MB progression.

Recently, we developed a novel 3D 384-well agar colony formation assay for MB cells of molecular subgroup 3. Using this assay, we screened a panel of plasma membrane calcium channel modulators and identified modulators of the T-type voltage-gated channels, mibefradil and NNC-55–0396, as selective growth inhibitors of MB cells [9]. Intriguingly, previous studies demonstrated that mibefradil suppressed the growth and stemness of glioblastoma stem-like cells and enhanced their sensitivity to temozolomide chemotherapy [10]. Mibefradil is a selective inhibitor of CaV3 T-type Ca^2+^ channel and was originally approved for treatment of hypertension and angina pectoris. Mibefradil was withdrawn from the market in 1998 due to potential interactions with other drugs frequently taken by patients with cardiovascular diseases. Mibefradil, in recent years, gained interest as a potential anti-cancer agent. Indeed, it recently successfully completed a phase I trial for recurrent high-grade glioma. Together with temozolomide, it was well-tolerated in patients with no signs of toxicity (ClinicalTrials.gov identifier NCT01480050 and publication [11]). NNC55-0396 (NNC) is an analogue of mibefradil with higher blood–brain-barrier permeability [12, 13]. In comparison to mibefradil, NNC also shows lower non-specific effects on L-type calcium channels [12, 13] and cytochrome P450 [13]. These properties suggest that NNC or similar derivatives may hold promise for clinical development and assessment of its effect in relevant cancer models is warranted.

The current study assessed the effect of mibefradil and NNC on MB cells and evaluated their mechanism of action. For the first time, this study shows that CaV3 inhibitors, mibefradil and NNC, induce apoptosis that is associated with altered metabolic activity in MB cells. This work provides new insights into the mechanism of action of mibefradil and NNC, and may pave the way to test these molecules and/or their analogues in in vivo pre-clinical models to assess their suitability as a potential treatment options for MB.

## Materials and Methods

### Cell Culture

MB cell lines, D341 Med (HTB-187™), CHLA-01-MED (CRL-3021™) and CHLA-01R-MED (CRL-3034™), were purchased from the American Type Culture Collection (ATCC) and cultured according to the supplier’s recommendation. D341 cells were cultured in Minimum Essential Medium Eagle (EMEM-M0643, Life Technologies, CA, USA) supplemented with 20% foetal bovine serum (FBS). CHLA-01-MED and CHLA-01R-MED were cultured in Dulbecco’s Modified Eagle’s Medium/Nutrient Mixture F-12 Ham (D8900, Sigma-Aldrich, MO, USA) supplemented with B-27™ Supplement (Sigma-Aldrich, Ryde, NSW, Australia), 20 ng/mL basic FGF (Sigma-Aldrich) and 20 ng/mL EGF (Sigma-Aldrich). Cells were maintained at 37 °C in a humidified atmosphere of 5% CO2.

### Real-Time RT-PCR

Total RNA from D341, CHLA-01-MED and CHLA-01R-MED cells were isolated and purified using ISOLATE II RNA Mini Kit (Bioline, London, UK; BIO-52073). The purified RNA was reverse transcribed using SensiFAST™ cDNA Synthesis Kit (Bioline; BIO-65054) and T100™ Thermal Cycler machine (Bio-Rad, Hercules, USA). The target cDNA was amplified using SensiFAST™ Probe No-ROX Kit (BIO-98020; Bioline) with the following primers: CACNA1G forward (5′-GGACTTCTCTTCATGTTGTTG-3′) and reverse (5′-GTCCTTCATAATGCCATTCC-3′); CACNA1H forward (5′-TATCTCGACCTCTTCATCAC-3) and reverse (5′-GACTTGGGTTGGTTATAGTG-3); CACNA1I forward (5′-CTTGGATTGTCATCTTCCAG-3′) and reverse (5′-TGAAGTTGTAGAAGGAGTGAG-3′); CACNA1S forward (5′-AGGAAAACTGTCTTTGGATG-3′) and reverse (5′-TGGATGATTTTGTTCAAGCC-3′); CACNA1C forward (5′-GGAGAGTTTTCCAAAGAGAG-3′) and reverse (5′-TTTGAGATCCTCTTCTAGCTG-3′); CACNA1D forward (5′-AAAATGGGCATCATTCTTCC-3′) and reverse (5′-AGTTTCATAATAGCGGGTTC-3′); CACNA1F forward (5′-CATTTTCACCATCCCAGAAG-3′) and reverse (5′-CTCATCTAGGTAGGAAAGCC-3′); RN18S1 forward (5′-ATCGGGGATTGCAATTATTC-3′) and reverse (5′-CTCACTAAACCATCCAATCG-3′) (Sigma-Aldrich, MO, USA). CFX Connect™ Real-Time PCR Detection System with Starter Package (Bio-Rad) was used to cycle and quantitative targets. The relative target quantity was determined using the comparative CT (ΔΔCT) method by normalising to 18S ribosomal RNA.

### WST-1 Assay

Metabolic activity of cells as a marker of cell viability and proliferation was assessed by WST-1 Cell Proliferation Assay Kit (10,008,883, Cayman Chemical, MICH, USA) according to the manufacture’s recommendation. Three MB cell lines, D341, CHLA-01 and CHLA-01R, were used in this assay. Briefly, cells were seeded at a density of 30,000 cells/well in 96-well plates. Twenty-four hours later, treatment was added as shown in the “Results” section. Assay reaction was assessed 24 or 72 h after treatment. For that, 10 μL of WST-1 reagent was added to each well for 4 h, and absorption was measured using a plate reader (Multiskan Go, Thermo Fisher Scientific, Scoresby, VIC, Australia). Drugs used, including mibefradil dihydrochloride hydrate (M5441), NNC55-0396 hydrate (N0287), verapamil hydrochloride (V4629), nifedipine (N7634), vincristine (V0400000) and lomustine (L5918), were purchased from Sigma-Aldrich.

### ATP Level and Protein Content

For the assessment of cellular ATP levels, a luminescent ATP assay was employed. 3 × 10^4^ cells were seeded in 100 µL per well in transparent 96-well plates and left to settle overnight. Subsequently, drug treatments were added to cells for 72 h. Cells were then transferred to Eppendorf tubes and centrifuged for 3 min at 200 g. Following media removal, cells were washed with sterile phosphate-buffered saline (PBS) followed by another centrifugation to obtain cell pellet. For cell permeabilisation, 40 μL of 0.5% Triton X-100/PBS was added to each tube for 10 min at room temperature. Ten microlitres of cell lysate was mixed with 90 μL assay buffer (300 μM d-luciferin, 5 μg/mL luciferase, 625 μM EDTA, 75 μM DTT, 6.25 mM MgCl_2_, 25 mM HEPES, 1 mg/mL BSA in PBS, pH 7.4) in white 96-well plates, followed by immediate measurement of luminescence using a plate reader (Fluoroskan Ascent, Thermo Fisher Scientific). Protein content from cell lysates (10 μL) was quantified using the DC Protein Assay (500–0116; Bio-Rad, CA, USA) as recommended by the manufacturer. Absorbance at 750 nm was measured using Multiskan Go Microplate Spectrophotometer (Thermo Fisher Scientific) and used for normalisation of ATP content in different treatments.

### Immunoblotting

For immunoblotting, 5 µg of total protein samples was resolved on a 10% Bis-Glycine-polyacrylamide gel and transferred onto Amersham™ Protran™ 0.2 μm Nitrocellulose (NC) blotting membrane (10,600,001, GE Healthcare). Proteins were detected using primary antibodies rabbit polyclonal PARP (9542S, Sigma-Aldrich) at a 1:1000 dilution, rabbit monoclonal caspase-3 (ab32042, Abcam, Cambridge, UK) at a 1:500 dilution and mouse monoclonal glyceraldehyde 3-phosphate dehydrogenase (GAPDH) (G8795, Sigma-Aldrich) at a dilution of 1:20,000 as loading control. Secondary antibodies included horseradish peroxidase (HRP)–conjugated goat anti-rabbit (170–6515, Bio-Rad, 1:3000 dilution) and goat anti-mouse IgG (170–6516, Bio-Rad, 1:3000 dilution). Anti-PARP and caspase-3 primary antibodies were incubated with the membrane at 4 °C overnight, while GAPDH and secondary antibodies were incubated at room temperature for 1 h. PageRuler™ Plus Prestained Protein Ladder (26,620, Thermo Fisher Scientific) was used to ease the detection of the molecular sizes (in kDa) of protein bands. Amersham™ ECL™ Prime Western Blotting Detection Reagent (RPN2236, GE Healthcare) was used to detect proteins. Digital images were recorded using the Amersham™ Imager 600 (29,083,461, GE Healthcare). Density of bands was quantified using Image Lab™ Software (version 6.0.1, Bio-Rad) and normalised to GAPDH.

### Sphere Generation

Spheroid generation was carried out by seeding CHLA-01R cells at 1 × 10^4^ cells per well in 200 μL volume in round bottom 96 Ultra-Low Attachment Microplate (7007, Corning, MA, USA). Spheroid formation was initiated by centrifugation of the plate at 1000 g for 10 min and the plate was incubated at 37 °C and 5% CO_2_ for 5 days until spheres were formed.

### Propidium Iodide (PI) Incorporation and Dual Live/Dead Viability Assays

To assess cell membrane integrity, as a marker of cell death, PI fluorescent staining was performed using PI dye. To stain MB spheres, 100 μL of media was removed from each well and PI at a concentration of 40 μg/mL in PBS (100 μL) was added to the treated spheres and analysed under a fluorescent microscope (IN Cell Analyzer 2000, GE Healthcare, IL, USA). ImageJ 1.49q software (NIH, Bethesda, MD, USA, website: https://imagej.nih.gov/ij/) was used to analyse PI incorporation by measuring the signal intensity of the sphere from the images. For live/dead dual staining, treated spheres were dissociated into single-cell suspension using Accutase solution (A6964, Sigma-Aldrich). Subsequently, cells were washed with PBS and stained with 3 μM calcein-AM (Thermo Fisher Scientific) and propidium iodide (5 μM) followed by incubation for 30 min. One hundred microlitres of cell suspension was transferred to black plates and imaged with fluorescent microscope (IN Cell Analyzer 2000) using excitation of 475 nm/emission of 511 nm for calcein-AM and excitation of 542 nm/emission of 620 nm for PI. Generated images were automatically analysed using IN Carta Image Analysis Software (GE Healthcare).

### Nuclear Morphological Staining

MB cells were seeded at 50,000 cells per millilitre in 24-well plates. Cells were treated with NNC or vehicle for 72 h. Subsequently, cells were stained with 5 µM Hoechst 33,342 (Thermo Fisher Scientific) for 30 min at 37 °C and examined under a fluorescence microscope (Nikon ECLIPSE 50i, Tokyo, Japan).

### Assessment of Mitochondrial Membrane Potential

Detection of altered mitochondrial membrane potential was performed using 5,5′,6,6′-tetrachloro-1,1′,3,3′-tetraethylbenzim-idazolylcarbocyanine iodide (JC-1) Mitochondrial Membrane Potential Assay Kit (ab113850, Abcam) according to the manufacturer’s instructions. Briefly, MB cells were washed twice and incubated with the assay solution containing JC-1 dye (10 μM) for 30 min. Cells were then washed, suspended in assay buffer (supplemented with 5% FBS) and seeded at 6 × 10^4^ in 50 μL in a black 96-well plate (655,090, Greiner, Germany). Fifty microlitres of 2 × concentration of treatments, including different concentrations of mibefradil, NNC and carbonyl cyanide 4-trifluoromethoxy phenylhydrazone (FCCP) as a positive control, or no treatment control, was added to cells followed by incubation at 37 °C for 6 h. Cells were imaged using IN Cell Analyzer 2000 (excitation wavelength used was 475 nm and emission wavelengths were 511 nm and 587 nm for the monomer and the aggregates of JC-1 molecules, respectively). Images were analysed using IN Carta Image Analysis Software (GE Healthcare).

### Mitochondrial Superoxide Production

To measure mitochondrial superoxide production, black 96-well plates (655,090, Greiner) were coated with poly-L-lysine (P4832, Sigma-Aldrich, Australia) for 60 min (1:1 in PBS, pH 7.4, 50 µL/well) before 5 × 10^4^ cells were seeded in 100-µL media per well and left to adhere for 1 h. Treatments (DMSO control and 3.5, 7.5 and 10 µM of NNC) were added for 6 h before MitoSOX Red (M36008, Thermo Fisher Scientific) at a concentration of 5 µM was added for 30 min. Fluorescence (Ex/Em 510/580 nm) was quantified using a plate reader (Tecan Infinite M Nano, Tecan Austria GmbH).

### Measurement of Cytosolic-Free Ca^2+^

Cytosolic-free Ca^2+^ levels were measured using Ca^2+^ indicator Fluo-4 (Fluo-4 NW Calcium Assay Kit, F36206, Invitrogen) according to the manufacturer’s protocol. Briefly, MB cells D341 and CHLA-01 were plated at 7.5 × 10^4^ in 100 µL per well in a black plate 96-well plate (655,090, Greiner) coated with poly-L-lysine (P4832, Sigma-Aldrich). After overnight incubation, cells were washed twice with the assay buffer (1.26 mM calcium chloride, 0.49 mM magnesium chloride, 0.40 mM magnesium sulphate, 5.33 mM potassium chloride, 0.44 mM potassium phosphate monobasic, 4.16 mM sodium bicarbonate, 137.9 mM sodium chloride, 0.33 mM sodium phosphate dibasic anhydrous) and loaded with the dye solution for 50 min at 37 °C and 10 min at room temperature. Dye solution was removed, and cells were washed with the assay buffer (same as above excluding calcium chloride). For treatment, NNC (3.5–20 µM) in 0.5 mM BAPTA (B1214, Thermo Fisher Scientific) was added for 10 min in Ca^2+^ free buffer. Fluorescence (excitation at 475 nm and emission at 525 nm) was measured at 20 °C using a plate reader (Tecan Infinite M Nano, Tecan Austria GmbH).

### Proteomic Analysis

MB cell line, CHLA-01, was seeded at 3 × 10^5^ cells per well in 12-well plate. After overnight incubation, cells were treated with different concentrations of mibefradil (0, 3.5 and 7.5 μM) for 72 h. Cells were washed with PBS and lysed using chilled lysis buffer, 100 mM NaCl, 1% IGEPAL®, 0.5% sodium deoxycholate, 50 mM Tris (pH 8.0), 1 × protease inhibitor (Roche Diagnostic GmbH, Germany) and 1 × phosphatase inhibitor (Roche Diagnostic GmbH, Germany). Protein concentrations were determined using Bio-Rad DC Protein Assay as described above.

Aliquots of 30 μg protein were sequentially reduced using 10 mM DTT overnight at 4 °C, alkylated using 50 mM iodoacetamide for 2 h at ambient temperature and then digested with 1.2 μg proteomics-grade trypsin/LysC (Promega) according to the SP3 protocol [14]. Digests were halted by the addition of TFA to 0.1% and peptides were collected by centrifugation at 21,000 × g for 20 min. Samples were desalted using ZipTips (Merck) according to the manufacturer’s instructions. Proteomic analysis deployed a combination of high-pH reversed phase microflow HPLC peptide fractionation and data-dependent acquisition (DDA) to generate a project-specific spectral library, followed by data-independent acquisition (DIA) for individual samples. Detailed method for LC/MS using a Q-Exactive HF and Ultimate 3000 RSLCnano LC/MS system (Thermo Scientific, Massachusetts, USA) is described in Espejo et al. [15].

DDA- and DIA-MS raw files were processed using Spectronaut software (version 13.12, Biognosys AB, Switzerland). The project-specific library was generated using the Pulsar search engine to search DDA MS2 spectra against the UniProt Homo sapiens protein sequence database using Biognosys (BGS) software factory settings, including N-terminal acetylation and methionine oxidation as variable modifications and cysteine carbamidomethylation as a fixed modification, up to two missed cleavages allowed and peptide, protein and PSM thresholds set to 0.01. Retention time alignment was based on the high precision IRT concept [16].With the exception that single-hit proteins were excluded, BGS factory settings were also used for relative quantitation between samples using the MaxLFQ algorithm (in Spectronaut version 15) for protein label-free quantitation and global normalisation based on median MS2 intensity values.

### Statistics and Bioinformatics Analysis

Proteins identified as candidates in Spectronaut (Supplemental Table S1) were imported into Perseus software (http://coxdocs.org/doku.php?id=perseus:start) for further processing. Label-free quantitation (LFQ) values were first log2 transformed and proteins with missing values were removed before using an unpaired *t* test for statistical analysis. Proteins with altered expression of ± 1.5 fold change and false discovery rate (FDR)-adjusted *p* values below 0.05 compared to the control (Supplemental Table S2) were considered significant and selected for the enrichment analysis. Statistical overrepresentation test of significantly altered proteins was conducted using PANTHER online tool [17] with GO Biological Process annotation. Statistical analysis was performed using GraphPad Prism (version 8.0, GraphPad Software, Inc., San Diego, CA). Details of statistical analyses are provided in the corresponding figure legends.

## Results

Given our previous studies suggesting the importance of CaV3 channels in MB [8, 9], in this study, we aimed to examine the effect of two T-type calcium channel inhibitors, mibefradil and NNC, on MB cells and evaluate their possible mechanism of action in group 3 (D341) and group 4 (CHLA-01 and CHLA-01R) MB cell lines.

**Fig. 1:**
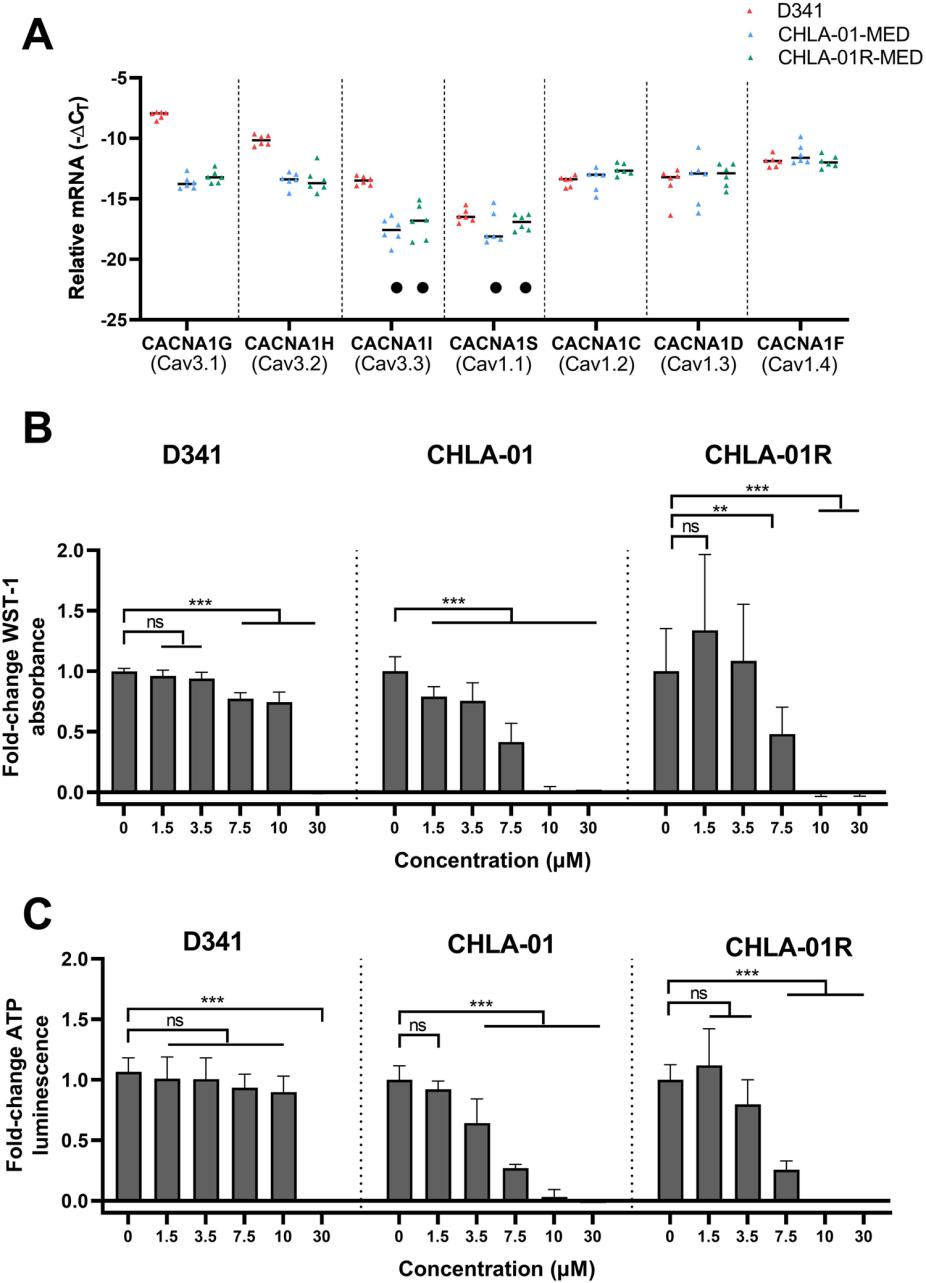
**A** Relative mRNA expression (− ΔCt) of seven CaV channels in three MB cell lines. (● denotes a target below the limit of detection (Ct > 35) in one or more replicates of samples in each cell line). Bar represents the mean of triplicates. **B**, **C** Mibefradil induces toxicity in MB cells. D341, CHLA-01 and CHLA-1R cells were treated with mibefradil for 72 h and subsequently assessed for toxicity by (B) WST-1 and (C) ATP assays. Data of each concentration was standardised to the non-treated control (0.1% DMSO) as fold change. Data expressed as mean ± standard deviation from three independent experiments with three replicates each. ns, not significant (*p* > 0.05), **p < 0.01, ***p < 0.001 (one-way ANOVA with Dunnett multiple comparisons test compared with the non-treated 0 control group)

We first analysed the gene expression of T- and L-type calcium channels in three MB cell lines used in this study (D341, CHLA01-MED and CHLA-01R-MED) (Fig. 1A). All the examined MB cell lines expressed T-type channels A1G and A1H and L-type channels A1C, A1D and A1F; however, only group 3 D341 cells showed detectable mRNA expression for T-type A1I and L-type A1S channels. Interestingly, all T-type channels are higher in D341 cells compared to CHLA-01-MED and CHLA-01R-MED cells.

To assess the effect of mibefradil on MB cell proliferation and ATP production, we treated three MB cell lines (D341, CHLA-01 and CHLA-01R) with increasing concentrations of mibefradil and the toxicity was assessed using WST-1 and ATP assays. Mibefradil treatment led to increased toxicity in all three cell lines in a concentration-dependent manner in both WST-1 (Fig. 1B) and ATP (Fig. 1C) assays as endpoints. This toxicity was more pronounced in CHLA-01 cells.

**Fig. 2:**
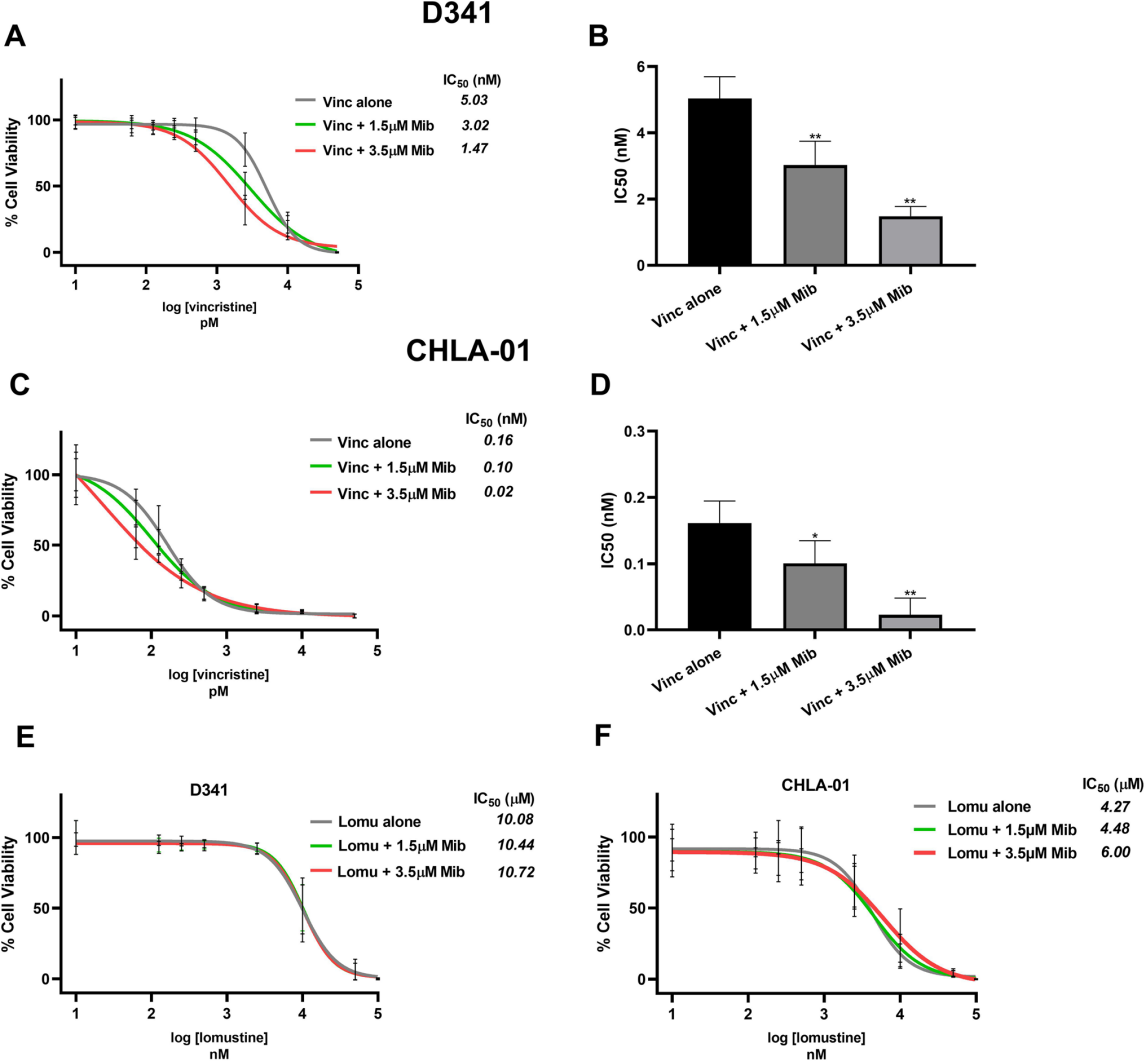
Cytotoxic effects of mibefradil in combination with chemotherapeutics. Concentration response curve and IC_50_ values of vincristine alone or in combination with mibefradil in **A** D341 and **C** CHLA-01. Concentration response curve and IC_50_ values of lomustine alone or in combination with mibefradil in **E** D341 and **F** CHLA-01 cells. Results for each concentration were standardised to the non-treated control and expressed as per cent control. **B**, **D** IC_50_ statistical data comparison in (B) D341 and (D) CHLA-01. All the values are expressed as mean ± standard deviation of three independent experiment; *n* = 3, one-way ANOVA, with Dunnett multiple comparisons test compared with Vinc alone group, *p < 0.001 and **p < 0.0001. Mib, mibefradil. Vinc, vincristine. Lomu, lomustine

Combination therapy is one approach to enhance the effect of current treatment options (e.g. chemo-/radiotherapies) and/or for dose reductions to reduce side effects. We, therefore, explored the effect of mibefradil co-treatment with the clinically used MB chemotherapeutic agents, vincristine and lomustine in D341 and CHLA-01 cells. Low mibefradil concentrations by themselves did not significantly affect MB growth. However, combined with vincristine, mibefradil improved the ability of vincristine to suppress MB cell growth. Indeed, combining vincristine with 3.5 µM mibefradil reduced the IC_50_ of vincristine around 3.5 times in D341 (from 5.03 to 1.47 nM, Fig. 2A–B) and 8 times in CHLA-01 cells (from 0.16 to 0.02 nM, Fig. 2C–D). Intriguingly, this enhanced effect was not seen with lomustine (Fig. 2E–F).

**Fig. 3:**
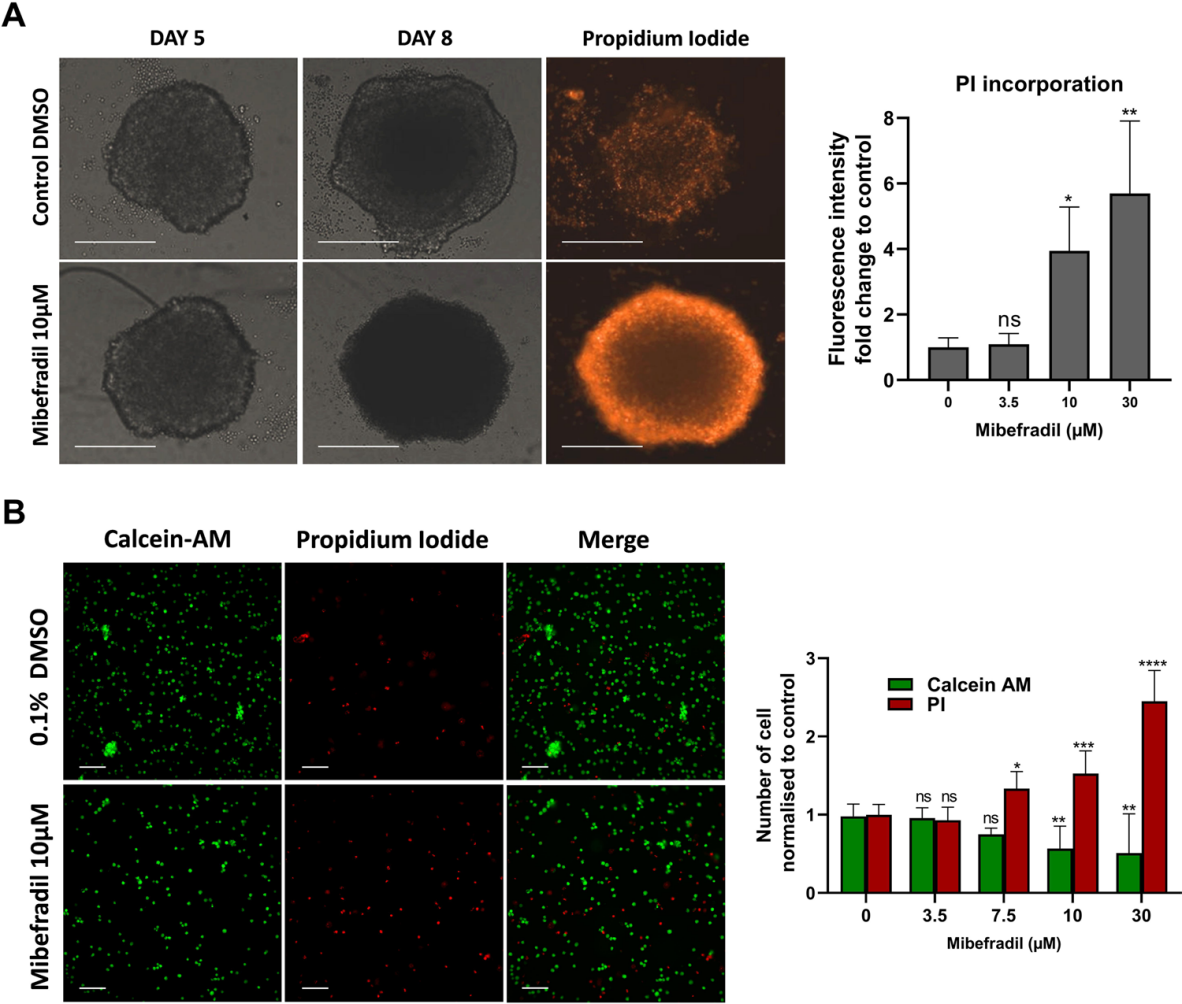
Mibefradil induces cell death in MB spheres. **A** Left panel: representative bright field and fluorescent images of CHLA-01R cell spheres at before (day 5) and after (day 8) treatment with mibefradil (10 µM) or DMSO control. The scale bar is 300 μm. Right panel: quantitative analysis of PI fluorescence intensity of DMSO control and increasing concentrations of mibefradil. Results for each concentration were standardised to the non-treated control and expressed as fold change**.** Data represents mean ± standard deviation from three independent experiments with three replicates each. ns, not significant (*p* > 0.05), *p < 0.01, **p < 0.001 (one-way ANOVA with Dunnett multiple comparisons test compared with the non-treated 0 control group). **B** Live/dead analysis of MB spheres treated with different concentrations of mibefradil. Generated spheres were grown for 5 days and treated for 3 days. Single-cell suspension from dissociated spheres was stained with calcein-AM/propidium iodide and imaged using IN-Cell 2200 analyzer (20 × magnification). Left panel: exemplary fluorescence images. Right panel: quantitative analysis of acquired images using IN Carta image analysis software. The scale bar is 60 μm. Results for each concentration were standardised to the non-treated control and expressed as fold change. Data expressed as mean ± standard deviation from three independent assays with three replicates each. ns, not significant (*p* > 0.05), *p < 0.05, **p < 0.01, ***p < 0.001, ****p < 0.0001 (two-way ANOVA with Dunnett multiple comparisons test compared with the non-treated control group)

Since at this point it was unclear if the observed toxicity represented metabolic toxicity or cell death, we assessed whether mibefradil induces cell death in MB cells. Treatment of CHLA-01R cell spheres with increasing concentrations of mibefradil induced cell death as indicated by propidium iodide (PI) staining (Fig. 3A). In a follow-up experiment, to ensure homogeneous distribution of the fluorescent dye to cells, subsequent to drug treatments, spheres were dissociated into single-cell suspensions. Mibefradil induced a concentration-dependent decrease in calcein-AM and a simultaneous increase in PI staining, indicative of induction of cell death (Fig. 3B).

**Fig. 4:**
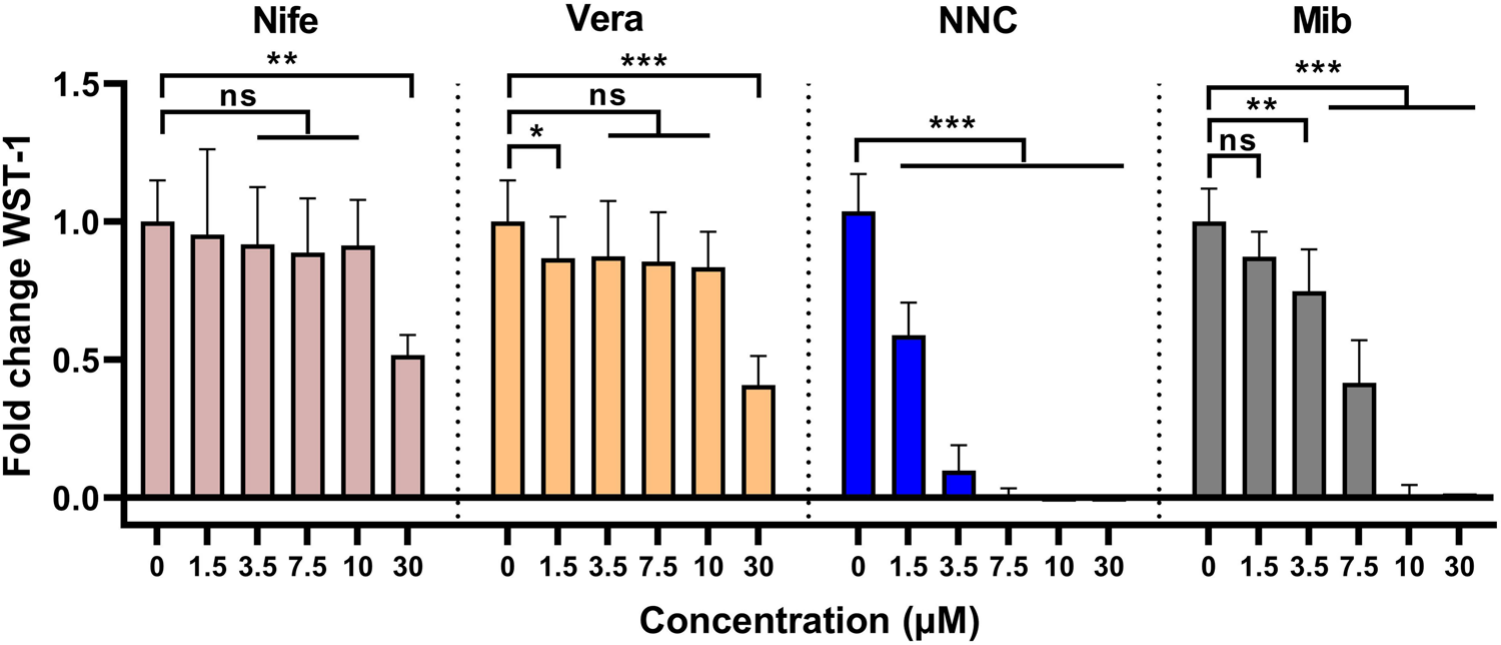
Blockade of T-type, but not L-type Ca^2+^ channels, reduces cell viability in MB cells. Viability of CHLA-01 cells, measured by WST-1 assay, treated for 72 h with increasing concentrations of Ca^2+^ channel antagonists. Results for each concentration were standardised to the non-treated control and expressed as fold change. Data expressed as mean ± standard deviation from three independent assays with three replicates each. ns, not significant (*p* > 0.05), *p < 0.05, **p < 0.001, ***p < 0.0001 (two-way ANOVA with Dunnett multiple comparisons test compared with the non-treated 0 control group). Nife, nifedipine. Vera, verapamil. Mib, mibefradil

**Fig. 5:**
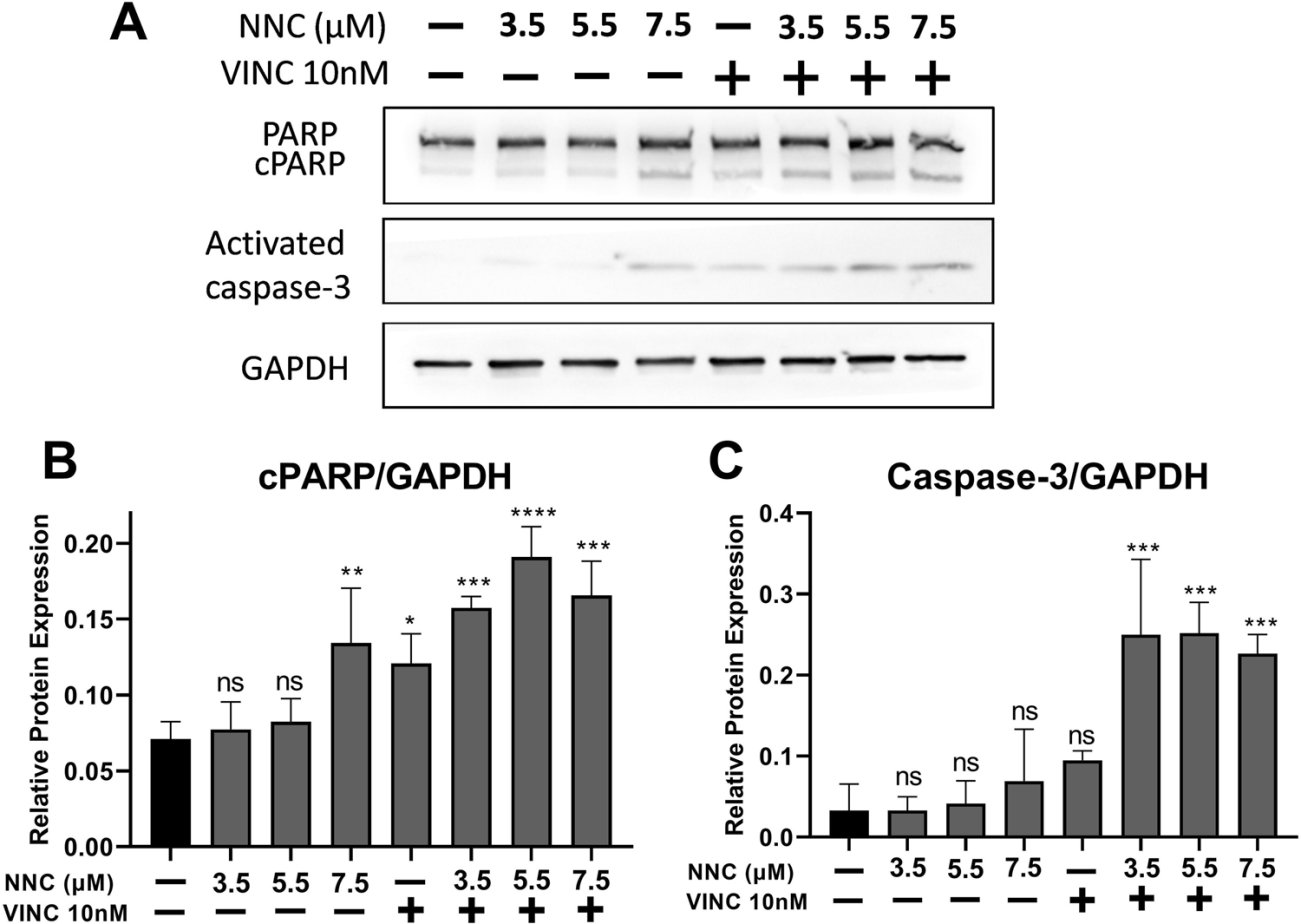
Effect of NCC on apoptotic cell death. D341 cells were treated for 12 h with different concentrations of NNC (0–7.5 µM) or vincristine (Vinc, 10 nM) alone or in combination. **A** Representative immunoblot of the cleaved PARP (cPARP), caspase-3 and GAPDH. Densitometric analysis of total **B** cleaved PARP (cPARP) and **C** activated caspase-3 (both normalised to GAPDH as loading control) (ns, not significant *p* > 0.05, *p < 0.05, **p < 0.01, ***p < 0.001, one-way ANOVA, with Dunnett multiple comparisons test compared with control (c) group), *n* = 3. Bars present mean ± standard deviation from three independent experiments

Mibefradil, a relatively selective T-type Ca^2+^ antagonist, has a metabolite that blocks L-type channels from within cells. Therefore, the antiproliferative action observed with mibefradil may be due to concurrent activity on the L-type Ca^2+^ channel. To assess whether the observed effect of mibefradil on MB cell growth was specific to T-type channel blockers, CHLA-01 cells were treated with mibefradil, its analogue, NNC55-0396 (NNC), or with L-type channel blockers, nifedipine and verapamil. L-type inhibitors did not affect MB cell growth to the same extent as T-type inhibitors (Fig. 4). Indeed, the L-type channel blockers only showed significant effects at the highest concentration tested (30 µM). Interestingly, NNC showed a greater inhibitory effect compared to mibefradil, with concentrations as low as 1.5 µM showing a significant effect (Fig. 4). Treatment of MB cells with NNC also concentration-dependently reduced constitutive Ca^2+^ influx, as well as store-operated Ca^2+^ entry (Fig. S2), indicating that CaV3 proteins are active Ca^2+^ channels in MB cells. Although CaV3 inhibition resulted in cell death, the mechanism was unclear from this data, which cell death mechanism [18] was present. Apoptosis is a key mechanism of cell death that has been utilised as a target of many treatment strategies [19]. To explore whether the observed cell death was via apoptosis, the expression of two apoptotic markers, caspase-3 and cleaved poly (ADP-ribose) polymerase (PARP), was assessed after treatment of MB cells with NNC alone or in combination with vincristine. NNC alone significantly increased PARP degradation only at the highest concentration tested (7.5 µM), an effect that did not reach statistical significance for activated caspase-3 expression (Fig. 5). Vincristine alone (10 nM) enhanced PARP cleavage but did not significantly activate caspase-3. In contrast, the combination of NNC and vincristine significantly and additively enhanced both PARP cleavage and caspase-3 activation to a much higher extent compared to vincristine or NNC alone (Fig. 5). This suggested that NNC and its combination with vincristine induce apoptotic cell death. Additionally, Hoechst 33,342 staining was used to determine the nuclear morphological changes via apoptosis. Hoechst 33,342 is a fluorescence cell-permeable stain used for labelling DNA. Treated cells exhibited characteristics of apoptotic cells such as nuclear fragmentation and chromatin condensation (Fig. S1).

**Fig. 6:**
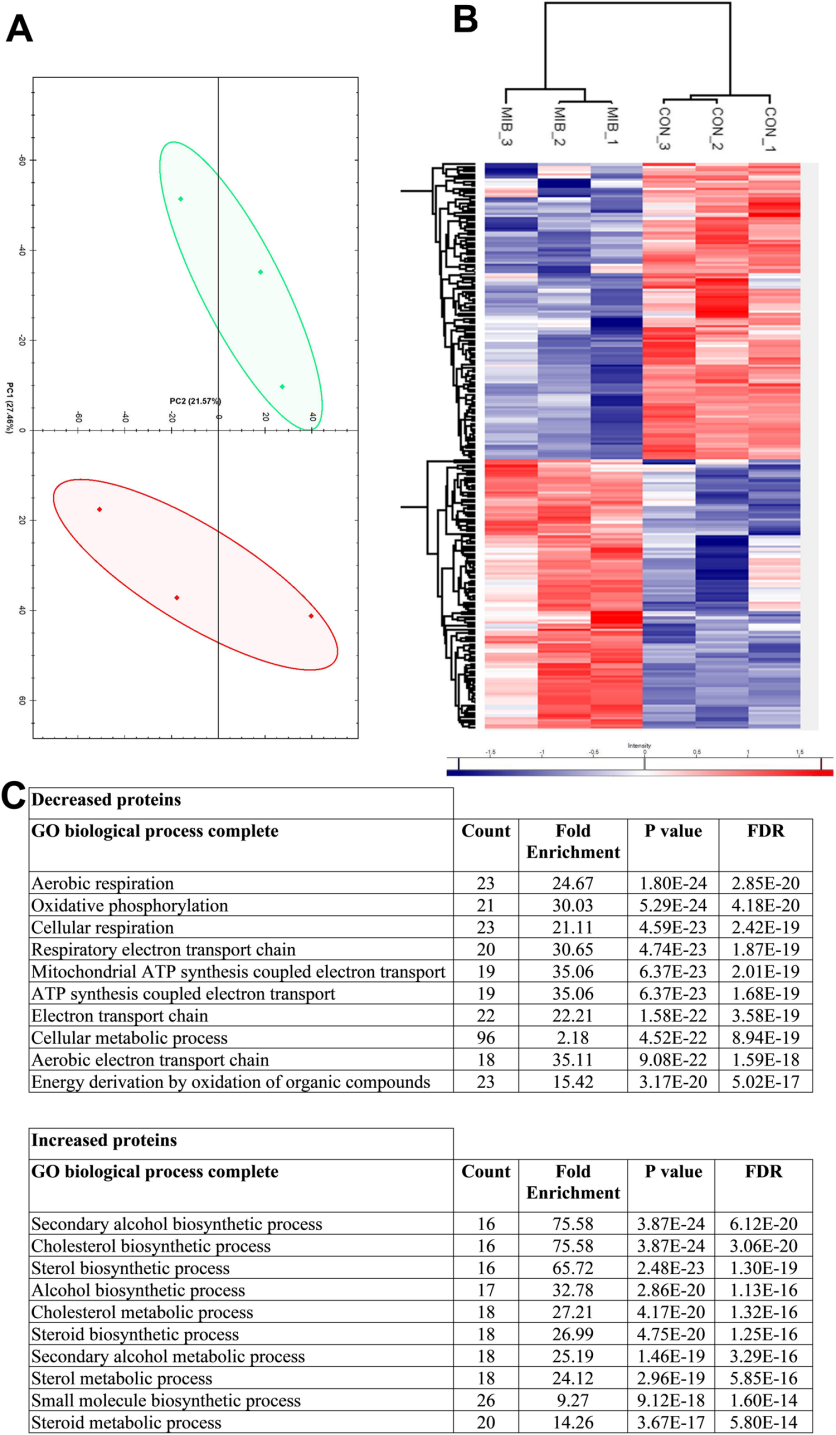
Proteomic analysis with mibefradil treatment. **A** Principal component analysis of the proteomics data showing control samples in red and treated samples in green, **B** hierarchical cluster analysis of significant proteins according to Z-scored LFQ values (C). Statistical overrepresentation test of proteins that were significantly increased (106 proteins, Table S2) or decreased (120 proteins, Table S2) by treatment of CHLA-01 MB cells with mibefradil (7.5 µM) for 72 h compared to the control. PANTHER online tool [17] and GO Biological Process annotation were utilised. Fisher’s exact test and false discovery rate (FDR) calculation were used for data analysis. Top 10 biological processes are shown. Complete list of the biological processes is shown in Table S3

Understanding the apoptotic mechanism is critical and can contribute to the development of drugs that target selective apoptotic pathways. Therefore, we used proteomics to further investigate the mechanistic effects of CaV3 inhibition in MB cells. In total, 4,819 protein groups were quantified across all samples. Principal component analysis of the LFQ data indicated clear separation of control and treated sample groups, although the individual samples did not cluster tightly (Fig. 6A). According to stringent criteria (FDR-adjusted *p* value < 0.05 and fold change > 1.5), 120 and 106 proteins were significantly decreased or increased by mibefradil, respectively (displayed as a heat map in Fig. 6B and listed in Table S2). Pathway enrichment analysis of the proteins significantly decreased by mibefradil treatment (Table S3) showed they mainly belong to mitochondrial oxidative phosphorylation (OXPHOS) and ATP generation processes (Fig. 6C). In contrast, proteins significantly increased with mibefradil were mainly associated with sterol and lipid metabolism processes (Fig. 6C). These data suggest an altered energy metabolism in MB cells as a result of mibefradil treatment.

**Fig. 7:**
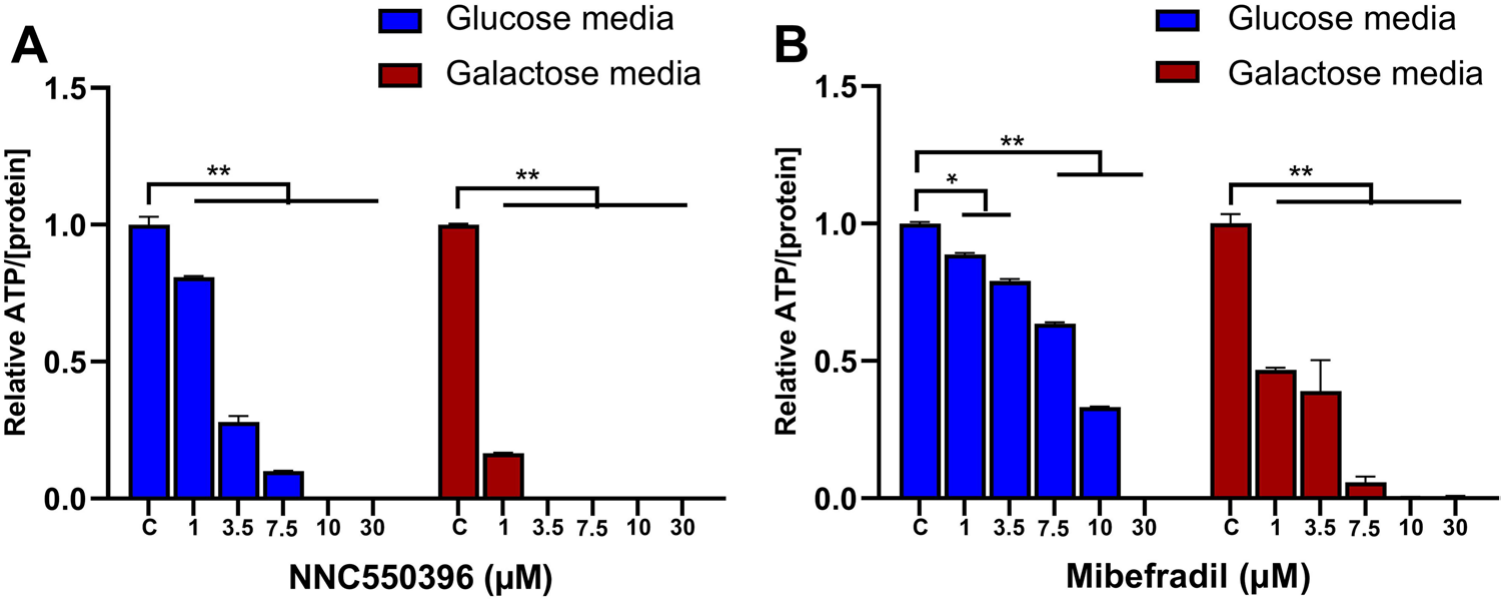
Effects of mibefradil and NNC on cellular ATP levels**. A**–**B** CHLA-01 cells were treated for 24 h with increasing concentration of (A) NNC and (B) mibefradil (0–30 µM) in the presence of glucose (blue) or galactose (red) media before ATP levels were quantified from cell lysate. All data were normalised to protein content. Data were expressed as mean fold change ± standard deviation from three independent assays with three replicates each, *p < 0.05, **p < 0.0001 (two-way ANOVA with Dunnett multiple comparisons test compared with the non-treated 0 control group)

**Fig. 8:**
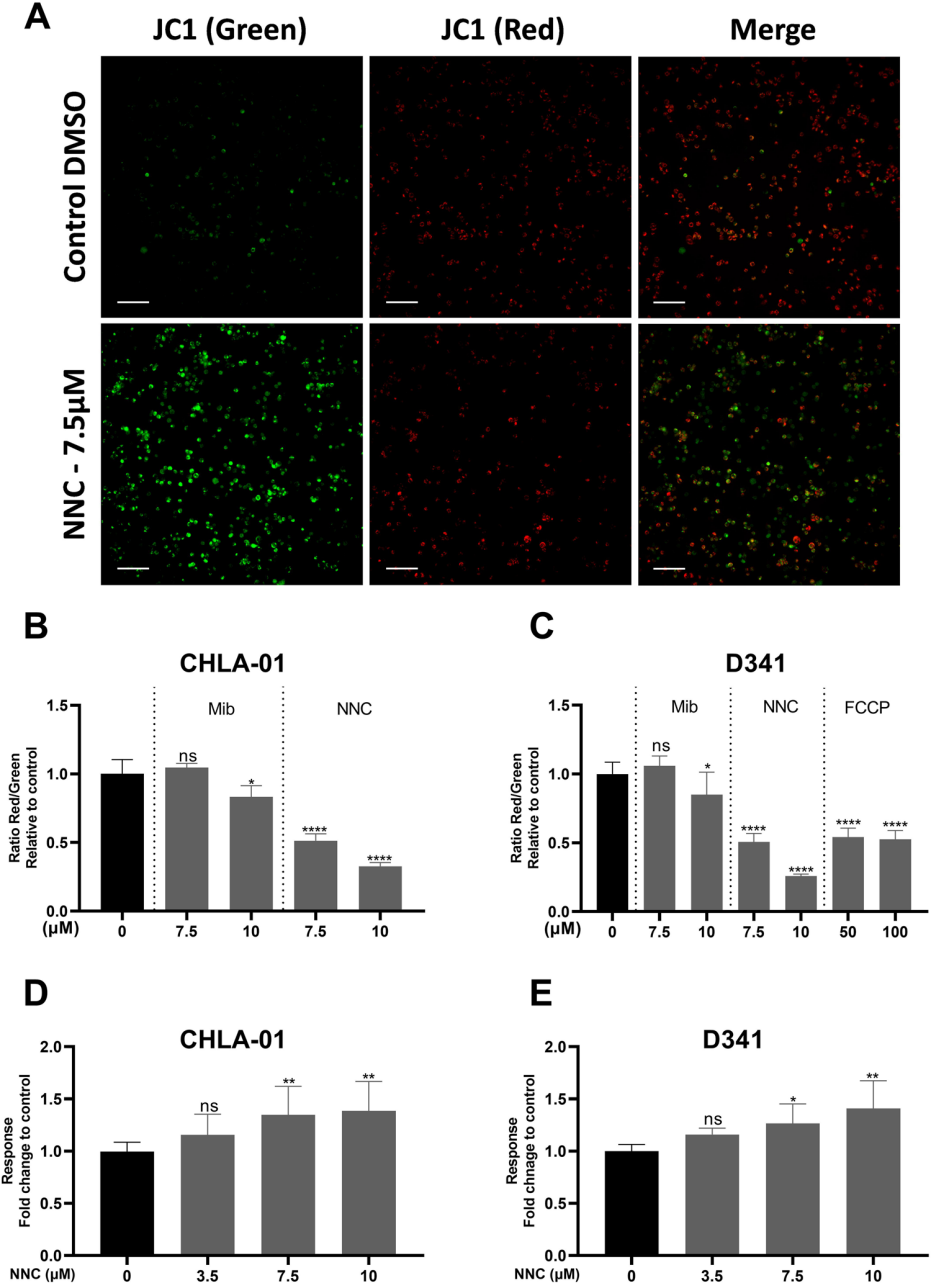
Effects of mibefradil and NNC on mitochondrial membrane potential and superoxide production. **A** Representative images of CHLA-01 cells stained with JC-1. Images show JC-1 green, JC-1 red and merge image of cells treated with NNC (7.5 µM) or vehicle control. (B, **C**) Mitochondrial membrane potential was quantified using JC-1 fluorescence. **B** CHLA-01 and **C** D341 MB cells were stained with JC-1 dye, treated with NNC, mibefradil or control, and analysed using high-content imaging using FITC and Cy3 filter sets. (D, E) Mitochondrial superoxide production was quantified using MitoSOX red. **D** CHLA-01 and **E** D341 MB cells treated with NNC, stained with MitoSOX dye and analysed using Fluorescence plate reader. Data were expressed as mean fold change ± standard deviation from three independent assays with three replicates each. ns, not significant (*p* > 0.05), *p < 0.05, **p < 0.01, ***p < 0.001, ****p < 0.0001 (one-way ANOVA with Dunnett multiple comparisons test compared with the non-treated 0 control group)

Given the suggested affected OXPHOS and ATP generation pathways by mibefradil, we next assessed intracellular ATP levels after treatment of cells with CaV3 inhibitors, mibefradil and NNC. To distinguish between ATP generated from glycolysis or OXPHOS, in addition to normal growth of cells in the presence of glucose, galactose-containing media was used [20]. Treatment of CHLA-01 cells with mibefradil or NNC reduced intracellular ATP levels in a concentration-dependent manner in both glucose and galactose media (Fig. 7A–B). This reduction was more pronounced in galactose media, suggesting potential effects on mitochondrial OXPHOS. This result supported the proteomics data and prompted further experiments to assess the effect of CaV3 inhibitors on mitochondrial function. To further investigate the effect of mibefradil and NNC on mitochondrial function, mitochondrial membrane potential was assessed. Accumulation of the polymeric form of JC-1 (red fluorescent aggregates) indicates high uptake of the stain into mitochondria, corresponding to high mitochondrial membrane potential [21]. Treatment with 10 µM mibefradil resulted in a modest but statistically significant reduction in the membrane potential of CHLA-01 and D341 cells (Fig. 8B–C). NNC treatment significantly reduced mitochondrial membrane potential in both CHLA-01 and D341 cells at 7.5 and 10 µM in a concentration-dependent manner (Fig. 8B–C). Trifluoromethoxy carbonylcyanide phenylhydrazone (FCCP), an uncoupler of the mitochondrial OXPHOS, was used as a positive control. FCCP suppressed mitochondrial membrane potential in D341 cells, but not to the same extent compared to NNC (Fig. 8C). These data suggest that CaV3 inhibitors directly affect mitochondrial function as a potential mechanism to induce cell toxicity by an apoptotic mechanism. To further assess the observed toxicity of the test compounds on mitochondria, mitochondrial superoxide production was measured. Consistent with JC-1 assay results, the data shows that production of superoxide is significantly enhanced with NNC treatment in a concentration-dependent manner confirming mitochondrial toxicity (Fig. 8D and E).

## Discussion

In the present study, we have identified the expression of T-type Ca^2+^ channels in medulloblastoma cell lines. We also identified and explored the selective toxicity of the CaV3 inhibitors, mibefradil and NNC, on MB cells. Our data demonstrated that both compounds lead to apoptotic cell death in MB cells, alone or in combination with the clinically used chemotherapeutic vincristine. This CaV3 inhibitor-induced cytotoxicity was associated with reduced intracellular ATP levels and mitochondrial function, indicated by a disruption of mitochondrial membrane potential. It is, however, still unclear how these compounds affect mitochondrial function in detail. Mitochondria play a critical role in the regulation of intracellular Ca^2+^ homeostasis. Mitochondria take up intracellular Ca^2+^ from the endoplasmic reticulum or from an influx of extracellular Ca^2+^ to prevent cytoplasmic Ca^2+^ overload. Conversely, this mitochondrial Ca^2+^ directly regulates mitochondrial function and affects ATP synthesis [22, 23].

Previous studies showed that T-type Ca^2+^ channel inhibitors induce apoptosis in leukaemia cells via Ca^2+^ overload from endoplasmic reticulum Ca^2+^ release [24]. However, our Ca^2+^ assays showed that this is not the case in MB cells as drug treatment only did not evoke ER Ca^2+^ release. Instead, it suppressed Ca^2+^ influx. Although Ca^2+^ overload is mainly associated with apoptosis, decreases in intracellular Ca^2+^ have been also linked to apoptosis [25].

The mechanism of apoptosis is highly complex and typically involves extrinsic or intrinsic pathways. Earlier apoptotic events involve caspase enzyme family activation, while later events include expression of phosphatidylserine on the external surface of the plasma membrane (measured by annexin V binding) and cleavage of PARP [26]. Nuclear fragmentation and condensation and membrane integrity loss (measured by PI incorporation) are usually end-stage apoptotic events [26, 27]. Given this timeline, we measured caspase-3 activation at 12 h and later events after 72 h of treatment. The apoptotic action of CaV3 inhibitor and its potential combination with vincristine was confirmed through western blotting for caspase-3 and its endogenous substrate PARP and through observing characteristics of apoptotic cells such as nuclear condensation and chromatin condensation after cells treatment with NNC.

Given the early disruption of mitochondrial membrane potential by CaV3 inhibitors, it will be of interest to explore mitochondrial Ca^2+^ levels after exposure of cells to mibefradil and NNC in future studies. This can be achieved using mitochondria-specific genetically encoded Ca^2+^ indicators [23] or small molecule fluorescent dyes that localise to mitochondria, such as Rhod-2 [28]. This study also identified that CaV3 inhibitors sensitised MB cells to the clinically used chemotherapeutic, vincristine. MB is a difficult cancer to treat, and chemotherapy treatment for MB is associated with considerable toxicities and potential long-term disabilities. Indeed, vincristine displays significant neurotoxic side effects in children including peripheral neuropathies and seizures [29, 30]. One possible approach to ameliorate side effects of vincristine is to decrease the total dose used. Our data showed enhanced effect of mibefradil with vincristine. This can be the first step to explore whether this novel combinatory strategy may provide increased treatment efficacy, while at the same time decreasing vincristine-associated adverse effects. Future studies are also warranted to understand the molecular mechanism of this enhanced effect, especially that it did not happen with the other chemotherapeutic agent used in the study, lomustine. Vincristine is known to induce cell death via cell cycle–dependent mechanisms, in particular in G1, where it binds irreversibly to microtubules and spindle proteins [31]. Calcium signalling also plays a critical role in the control of cell cycle through multiple checkpoints [32, 33]. Given this association, future studies should explore the effect of CaV3 inhibitors on cell cycle phases in combination with vincristine. Lomustine, on the other hand, is an alkylating agent that interferes with DNA replication by breaking DNA helix. It is a cell cycle non-specific agent, exerting its effect in all phases of the cell cycle, including G0 [34, 35]. This may explain why this agent did not show enhanced action when combined with T-type Ca^2+^ channel inhibitor.

This study did not assess whether the effect of mibefradil and NNC is mediated by a specific CaV3 channel. Mibefradil is a broad T-type calcium channel blocker and weak L-type calcium channel blocker [36], while NNC blocks CaV3.1 and CaV3.2 [12, 37]. Further studies using siRNA-mediated silencing are warranted to assess the effect of each of the CaV3 isoforms on MB growth and metabolic activities.

A growing number of studies reported that mibefradil and NNC-55–0396 could inhibit human cancer cell proliferation and induce cell apoptosis due to their ability to inhibit the function of T-type Ca^2+^ channels [38–40]. Mibefradil recently successfully completed a phase I trial for recurrent high-grade glioma. In combination with temozolomide, a dose of 100 mg of mibefradil four times daily was found to be tolerated and showed no toxicity by the patients (average plasma concentration was found to be 1.693 ± 0.287 μg/mL) (NCT01480050) [11]. In our study, 1.5 and 3.5 µM mibefradil, equal to 0.743 and 1.734 μg/mL respectively, were found to be significantly effective in enhancing the effect of vincristine against MB cells. In another phase I dose expansion trial, mibefradil was safely co-administered with hypofractionated radiation therapy over a 17-day period at 200 mg/day in patients with recurrent glioblastoma (NCT02202993) [41]. As discussed, NNC, an analogue of mibefradil, possesses multiple advantages compared to mibefradil that makes it attractive for potential future clinical trials. Our studies here showed that NNC is more effective than mibefradil in inducing toxicity in MB cells. This could be due to the enhanced selectivity of NNC on inhibition of T-type channels compared to mibefradil [12]. Future studies can work towards testing mibefradil and NNC in in vivo models of MB to assess their efficacy in suppression of tumour progression.

Overall, this study demonstrated that the CaV3 inhibitors, mibefradil and NNC, alter metabolism and induce apoptotic cell death in MB cells. Future studies need to test mibefradil and NNC, as well as their analogues in in vitro and in vivo models, to explore the possibility of using these compounds as potential future treatment strategies against MB.

## Supplementary Information

Below is the link to the electronic supplementary material.

Supplementary file1 (DOCX 3048 KB)

Supplemental Table S1 (XLSX 58.9 KB)

Supplemental Table S2 (XLSX 58.8 KB)

Supplemental Table S3 (XLSX 27.6 KB)

## Data Availability

The DIA-MS and DDA-MS raw data and Spectronaut pivot reports including the complete sets of identified peptides and proteins are available at ProteomExchange (dataset PDX026988) with reviewer login and password: reviewer_pxd026988@ebi.ac.uk; Pfc4cgjc.
