# Supplementary material for: T-Type Calcium Channel Inhibitors Induce Apoptosis in Medulloblastoma Cells Associated with Altered Metabolic Activity
Source: Mol Neurobiol. 2022 Mar 4;59(5):2932–45. doi: 10.1007/s12035-022-02771-0 (PMC9016057; doi:10.1007/s12035-022-02771-0)
Supplement: Supplementary file 1 — Supplementary file1 (DOCX 3048 KB) [file 12035_2022_2771_MOESM1_ESM.docx]

T-type calcium channel inhibitors induce apoptosis in medulloblastoma cells associated with altered metabolic activity

Mohammed Sedeeq, Ahmed Maklad, Taush Dutta, Zikai Feng, Richard Wilson, Taush Dutta, Nuri Gueven, Iman Azimi^1^

**Supplementary Figures**

**Figure S1.** Cellular and nuclear morphology of CHLA-01 cells treated with NNC (0 and 3.5µM) and stained with Hoechst 33342. (A) Exemplary images showing fragmented and condensed chromatin as indicated by arrow of MB cells. The scale bar is 60 μm. (B) Numerical data expressed as percent apoptotic cells between treated and non-treated group, Data points represent an average of two experiments (mean ± standard deviation).


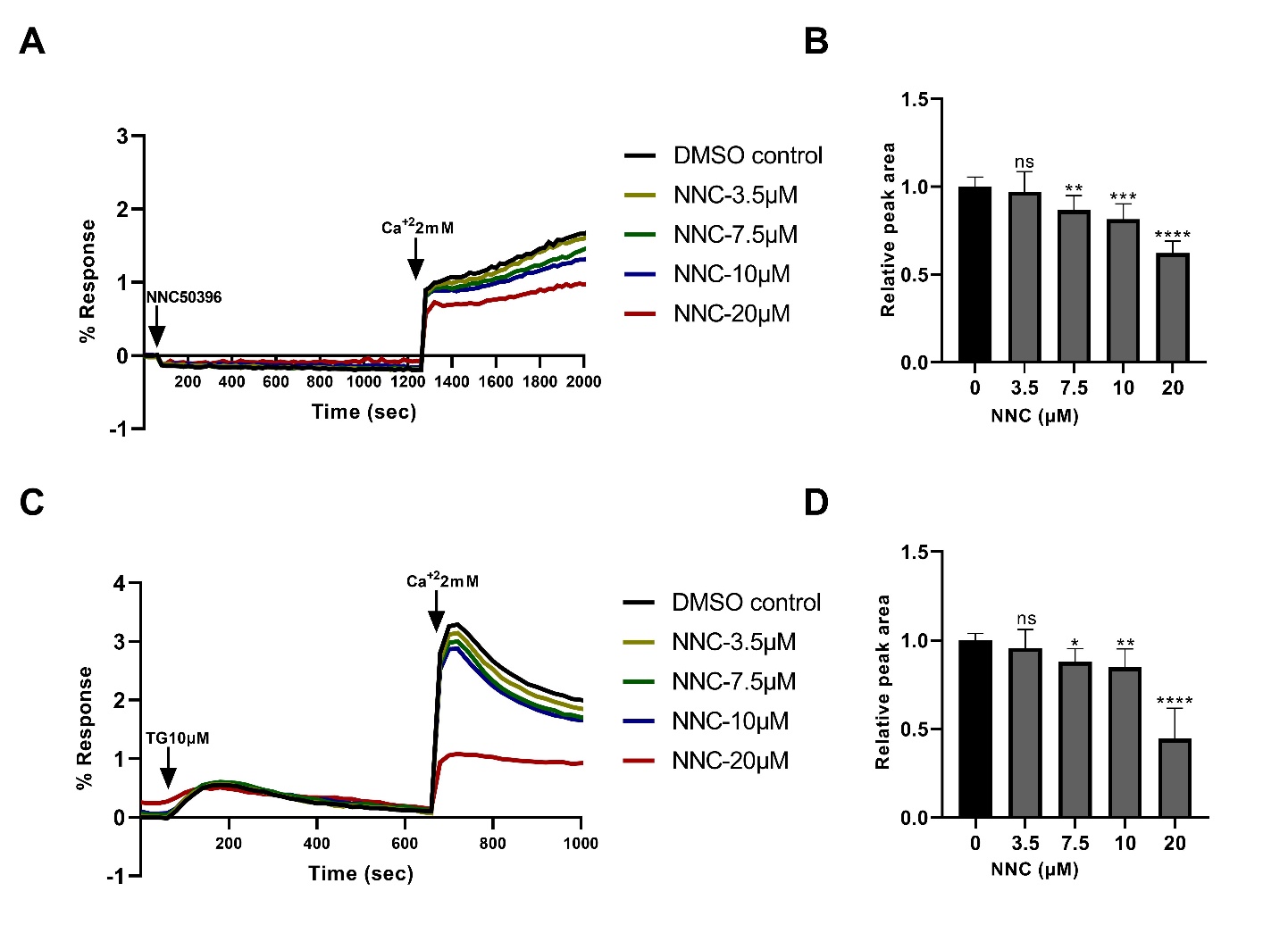


**Figure S2**. **Effects of T-type Ca^2+^ channel antagonist, NNC on intracellular Ca^2+^ levels in D341 cells.** (A, B) Assessment of constitutive Ca^2+^ entry in D341 cells. (A) MB cells were treated with NNC or control in Ca^+2^ free buffer followed by restoration of 2 mM Ca^+2^. (B) Quantification of relative peak area at 1240 to 2000 seconds. (C, D) Assessment of store operated Ca^2+^ entry in D341 cells (C**)** MB cells were treated with NNC or control in the absence of Ca^+2^ before the addition of 10 µM Thapsigargin (TG) and restoration of 2 mM Ca^+2^ (D) Quantification of relative peak area at 660 to 1000 seconds. Data expressed as mean (F-F_0_/F_0_) ± standard deviation from three independent experiments with three replicates each. ns = not significant (*p* > 0.05), * *p* < 0.01, ** *p* < 0.001, (one-way ANOVA with Dunnett multiple comparisons test compared with the non-treated 0 control group.
